# Body Composition and Dietary Intake of Women Attending an Infertility Clinic—Polish Observational Study

**DOI:** 10.3390/nu16234070

**Published:** 2024-11-27

**Authors:** Adriana Szulińska, Barbara Grzechocińska, Agnieszka Bzikowska-Jura

**Affiliations:** 1 Laboratory of Human Milk and Lactation Research, Department of Medical Biology, Medical University of Warsaw, 00-575 Warszawa, Poland; abzikowska@wum.edu.pl; 2 1st Department and Clinic of Obstetrics and Gynecology, Medical University of Warsaw, 02-015 Warszawa, Poland; barbara.grzechocinska@wum.edu.pl

**Keywords:** diet, protein intake, female infertility, body composition, obesity

## Abstract

Background and objectives: We aimed to assess the body composition and dietary intake of female patients attending one of the Polish infertility clinics. Additionally, we evaluated if there were any relationships between dietary intake and body composition parameters. Methods: The study involved 51 women who met the inclusion criteria. For the nutritional assessment, we used 3-day dietary records. Weight, height, waist and hip circumferences, and body composition were assessed. The participants were divided into three groups, with low (I, *n* = 12), normal (II, *n* = 27), and high (III, *n* = 12) fat tissue content, and then compared in terms of dietary intake. Results: The lowest protein intake per kilogram of body weight was observed in group III (*p* < 0.001). In group I, we reported the highest consumption of plant protein in general (*p* = 0.03) and per kg of body weight (*p* < 0.001). Higher protein intake per kg body mass was associated with lower values of BMI (r = −0.681; *p* < 0.001), fat mass (r = −0.641; *p* < 0.001), waist–hip ratio (r = −0.391; *p* = 0.005), and abdominal fat index (r = −0.653; *p* < 0.001). Conclusions: Our findings suggest that targeted nutritional counseling focused on optimizing protein intake and emphasizing plant-based sources may improve body composition and potentially support fertility outcomes in women undergoing infertility treatment.

## 1. Introduction

Infertility is a common health problem of great importance, especially in developed countries. According to the World Health Organization (WHO) report presented in 2023, the estimated incidence of lifetime infertility is 17.8% and 16.5% in high-income and low-income countries, respectively [1]. Infertility is defined as a lack of pregnancy after at least 12 months of regular unprotected intercourse. Infertility can also be diagnosed when in one or both partners impaired reproductive functions are observed [2]. In 50% of couples’ infertility cases, the female factor is decisive [3]. Female infertility can be a result of impaired or absent ovulation, tubal dysfunction, decreased ovarian reserve, or uterine or cervical dysfunction [4]. Infertility may also occur in polycystic ovary syndrome, endometriosis as well as premature ovarian failure and primary ovarian insufficiency [2]. In about 15% to even 30% of cases, the exact cause of the problem cannot be indicated [2,4]. However, the lack of a diagnosis does not mean that a cause does not exist, but it proves that the available, standard diagnostic methods are insufficient to detect it [2].

Apart from health-related factors, environmental factors may also matter when the risk of infertility is considered. These include smoking, excessive alcohol and caffeine consumption, drug use, being overweight, risky sexual behavior, chronic stress, and exposure to toxic substances, which are recognized modifiable factors [2]. Obesity is associated with a higher risk of infertility, affecting the ovulation process. In turn, reducing body weight brings significant improvement and is strongly recommended for overweight women during infertility treatment [3]. Not only body weight is important for reproductive results but also the nutritional profile of the diet (e.g., type of dietary fatty acids and proteins, the glycemic index of the diet, and the intake of antioxidants) [5]. In both women and men, nutritional factors have been proven to be related to increased risk of infertility. Research shows that some dietary patterns may affect fertility-related factors. For example, the Mediterranean diet, rich in vegetables, fruits, olive oil, unprocessed carbohydrates, fatty fish, and lean meat products, has a beneficial effect on fertility by reducing the risk of obesity, insulin resistance, and metabolic disorders. In turn, the Western diet, rich in simple sugars and processed meat, impairs the hormonal balance and the formation of reproductive cells [6]. Lifestyle modification, including dietary factors, is an intervention that may positively impact reproductive health and is relatively low cost [3].

The present study aimed to assess the energy and macronutrient intake from the diet in a group of women visiting an infertility clinic and to find relationships between the intake of macronutrients and anthropometric parameters with a known impact on female fertility. Understanding the nutritional trends specific to this group, their effect on body composition, and the relationship between nutrient intake will determine the most appropriate direction for nutritional counseling for women with infertility.

## 2. Material and Methods

### 2.1. Study Design

This observational study was conducted at the Infertility Clinic at the University Center for Women’s and Newborn Health in Warsaw, Poland, from January 2022 to July 2023. Participants were recruited among women attending the clinic for routine gynecological or infertility-related appointments. Potential participants were approached by the study coordinator (ASz), who briefly explained the study’s purpose and provided written materials outlining the study details, inclusion criteria, and informed consent forms. Women who expressed interest and met initial eligibility criteria were scheduled for an in-depth interview with a clinical dietitian and provided written informed consent before participation.

The inclusion criteria were as follows: women over 18 years of age with a confirmed diagnosis of infertility (defined as failure to conceive after at least 12 months of regular, unprotected intercourse) and no previous pregnancies. Exclusion criteria focused on conditions with a direct impact on infertility, such as anatomical abnormalities of the reproductive organs (e.g., fallopian tube obstruction). However, metabolic conditions commonly associated with infertility, including polycystic ovary syndrome (PCOS) and insulin resistance, were not excluded, as these are prevalent within the infertility patient population and pertinent to understanding the relationship between dietary intake, body composition, and reproductive health.

### 2.2. Dietary Assessment and Body Composition Analysis

To assess women’s dietary habits and nutritional intake, we used a three-day dietary record. Information about all foods and beverages consumed by the study participants for three days before the face-to-face interview was collected. The interviews were performed by the main investigator—a clinical dietitian (ASz). Sizes of declared food portions were verified using the “Album of Photographs of Food Products and Dishes” from the National Food and Nutrition Institute [7]. Finally, collected data were used to estimate daily food consumption. The sizes of declared food portions and meals were changed to grams/mL before calculation. For the calculation of dietary intake, we used Dieta 6.0 Software (National Food and Nutrition Institute, Warsaw, Poland), a Polish reference method based on the Polish food composition database.

### 2.3. Anthropometric Measurements and Body Composition Analysis

Each woman was weighed, and her height was measured. Both anthropometric parameters were measured to the nearest ±0.1 kg/cm. The body mass index (BMI) was calculated as the ratio between the body weight and the height squared (kg/m^2^). Interpretation of this data followed the classification proposed by the WHO: <18.5 kg/m^2^, underweight; 18.5–24.99 kg/m^2^, normal weight; >24.99 kg/m^2^, overweight or obese [8]. Each woman’s waist circumference (at the narrowest point of the torso) and hip circumference (at the widest point at the buttocks level) were measured using an anthropometric tape measure. Then, the waist–hip ratio was calculated by dividing the waist circumference by the hip circumference. Additionally, after excluding contraindications (pacemaker, metal implants, stents, large implants, implanted devices that emit an electronic signal) whole-body impedance of the women was measured using the Tanita BC-1000 (Tanita, Tokyo, Japan), according to the manufacturer’s instructions. Before taking the BIA measurement, the women were instructed with the following guidelines: no large meals or caffeinated products 4 h before the analysis, no heavy exercise 12 h before the test, and consumption of liquids limited to 1% of body weight or two 8 oz. glasses of water 2 h before the test [9].

Obtained parameters included body weight, percentage of fat mass (FM), percentage of total body water, muscle mass, abdominal fat index, and basal metabolic rate (BMR). Based on the results from the body composition analysis, in particular the percentage of total fat mass, we assessed the nutritional status of each participant. The assumed ranges considered as underweight, normal weight, and overweight were as follows: for women aged 20–39 years (*n* = 48)—<21%, 21–33%, and >33% of fat mass, respectively, and for women aged ≥ 40 years (*n* = 3)—<23%, 23–34%, and >34% of fat mass, respectively (according to the device producer’s recommendations) [10].

### 2.4. Statistical Analysis

Analyzed variables are presented as the median followed by the interquartile range. The Kruskal–Wallis test was used to compare analyzed variables in 3 groups. Spearman or Pearson correlation coefficients were calculated depending on variable distribution (verified by the Shapiro–Wilk test). Statistical analyses were performed using R software (version 3.4.0; R Core Team, Vienna, Austria). R is a language and environment for statistical computing available from the R Foundation for Statistical Computing (Vienna, Austria).

## 3. Results

### 3.1. Characteristics of the Study Group

In total, the study involved 51 female patients from a Warsaw infertility clinic with a mean age of 33.7 ± 3.2 years. Most of the participating women had a normal body fat content (*n* = 27, 53%), and the same number (*n* = 12, 24%) had a low or high body fat content. We found no differences in terms of age. BMI values exceeding 25.0 kg/m^2^ were reported in 15 women (29%), and 8 women had a WHR (waist–hip ratio) ≥ 0.85 (16%). Data regarding age, anthropometric parameters and basal metabolic rate are available in Table 1.

The average duration of infertility in the group was 3.0 ± 1.7 years (in the case of 13 women, no clear answer was obtained). The shortest period of diagnosed infertility in this group was four months, and the longest was eight years. In the study group, 46 women took dietary supplements. The most common chronic disease in the group was hypothyroidism. Demographic and anamnestic characteristics of the group are presented in Table 2.

### 3.2. Dietary Intake

Table 3 shows the intake of energy and nutrients in groups I, II, and III. The groups with low, normal, and high total body fat content did not differ significantly regarding energy, fat (including individual types of fatty acids), or carbohydrate intake. Protein intake, when converted per kilogram of body weight, was the lowest in the group with a high fat tissue content (*p* < 0.001). The highest plant protein consumption was found in the women with the lowest body fat content (*p* = 0.03). The groups also differed significantly in their intake of plant protein per kg of body weight (*p* < 0.001). In group I, the consumption of dietary fiber was also the highest (*p* = 0.010).

The total protein intake was positively correlated with energy intake (r = 0.573; *p* < 0.001), animal protein intake (r = 0.848; *p* < 0.001), the ratio of animal to vegetable protein (r = 0.510; *p* < 0.001), and total fat intake (r = 0.397; *p* = 0.004) but also saturated (SFA) (r = 0.404; *p* = 0.003) and monounsaturated (r = 0.316; *p* = 0.024) fatty acids (MUFA) as well as eicosapentaenoic acid (EPA) (r = 0.332; *p* = 0.017), docosahexaenoic acid (DHA) (r = 0.293; *p* = 0.037), and a lower percentage of energy from carbohydrates (r = −0.322; *p* = 0.021). The lowest and highest total protein intakes in groups I, II and III were 42.2 and 98.3 g, 55.3 and 132.6 g, and 54.9 and 124.6 g, respectively.

A higher protein intake per kg body mass was associated with lower values of weight (r = −0.655; *p* < 0.001), BMI (r = −0.681; *p* < 0.001), fat mass (r = −0.641; *p* < 0.001), waist circumference (r = −0.528; *p* < 0.001), abdominal circumference (r = −0.608; *p* < 0.001), waist-hip ratio (r = −0.391; *p* = 0.005), and abdominal fat index (r = −0.653; *p* < 0.001). A higher protein intake per kg body mass was also associated with higher intake of dietary fiber (r = 0.344; *p* = 0.014) and animal protein (r = 0.523; *p* < 0.001). In 44 (86%) women, the protein intake in g/kg of body weight was >0.9 (while 0.9 g/kg of body weight is the Recommended Dietary Allowance (RDA) for protein intake according to Polish Nutritional Standards [11]). In groups I, II, and III such consumption occurred in 12 (100%), 27 (100%), and 5 (42%) women, respectively. In 7 women in group III, the protein intake in g/kg body weight was below 0.9 g/kg body weight.

Additionally, the more dietary energy that came from protein, the higher the intake of animal protein (r = 0.712; *p* < 0.001) and ratio of animal to plant protein (r = 0.633; *p* < 0.001). Moreover, a greater amount of energy from proteins correlated positively with the consumption of EPA (r = 0.410; *p* = 0.003) and DHA acids (r = 0.469; *p* = 0.001) and negatively with the consumption of total carbohydrates (r = −0.365; *p* = 0.008).

A higher ratio of animal to plant protein was associated with a higher intake of saturated fatty acids (r = 0.338; *p* = 0.015) and lactose (r = 0.568; *p* < 0.001) and a lower amount of energy from carbohydrates (r = −0.502; *p* < 0.001) and dietary fiber intake (r = −0.499; *p* < 0.001). An animal-to-plant protein ratio in the diet exceeding 1.0 occurred in 7 (58%), 22 (81%), and 10 (83%) in groups I, II, and III, respectively.

High consumption of animal protein correlated positively with the intake of total energy (r = 0.332; *p* = 0.017), total protein (r = 0.848; *p* < 0.001), protein per body mass (r = 0.522; *p* < 0.001), total fat (r = 0.300; *p* = 0.033), saturated fatty acids (r = 0.415; *p* = 0.002), EPA (r = 0.343; *p* = 0.014), and lactose (r = 0.519; *p* < 0.001). However, there was a significant negative correlation with the amount of energy intake from carbohydrates (r = −0.485; *p* < 0.001).

In the study group, higher consumption of plant protein correlated positively with higher consumption of polyunsaturated fatty acids (r = 0.408; *p* = 0.003), linoleic acid (r = 0.432; *p* = 0.002), carbohydrates (r = 0.539; *p* < 0.001), and dietary fiber (r = 0.827; *p* < 0.001).

**Figure 1:**
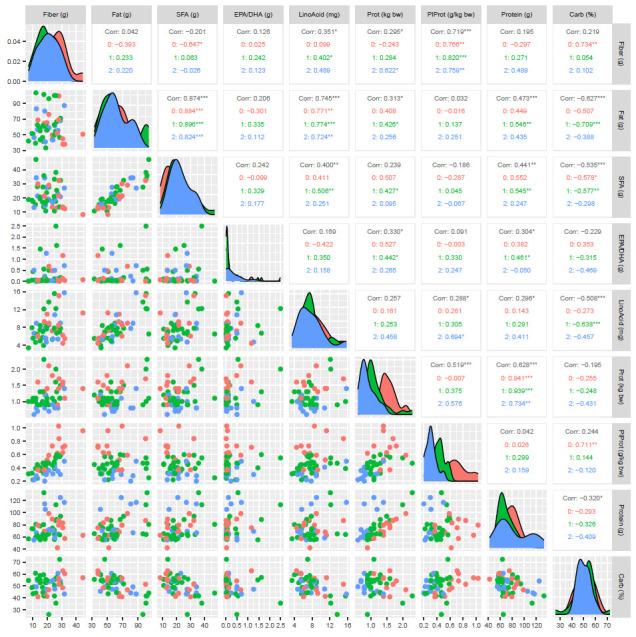
Correlations between the intake of individual nutrients. SFA—saturated fatty acids; EPA/DHA—eicosapentaenoic acid and docosahexaenoic acid combined; LinoAcid—linoleic acid; Prot—protein; PlProt—plant protein; Carb—carbohydrates; *** if the *p*-value is <0.001, ** if the *p*-value is <0.01, * if the *p*-value is <0.05.

Women with greater dietary fiber intake also consumed more polyunsaturated fatty acids (PUFA) (r = 0.321; *p* = 0.022), linoleic acid (r = 0.287; *p* = 0.041), and total carbohydrates (r = 0.450; *p* = 0.001). A graphical representation of the correlations between the intake of individual nutrients is shown in Figure 1.

In 12 (24%) women, energy intake from fat exceeded 35% (the highest recommended intake for this nutrient according to Polish Nutritional Standards [11]). For all groups, this excess was reported for 6 women (22%) in group II and 3 women (25%) in groups I and III. A fat intake below 20% of the energy value of the diet concerned 1 woman in group II. An SFA intake exceeding 10% of the dietary energy was noticed in 5 (42%), 19 (70%), and 8 (67%) women in groups I, II, and III, respectively. The intake of EPA and DHA was below 250 mg/d in 9 (75%), 21 (78%), and 8 (67%) women, respectively.

In 31 (61%) women, the energy intake from sugar exceeded 5%. For groups, I, II, and III such intake was reported in 8 (67%), 16 (59%), and 8 women (67%), respectively. The highest sugar consumption amounted to 17.7% of the daily energy intake. Table 4 shows the characteristics of nutrient intake in the study group compared with the Polish Nutritional Standards [11].

## 4. Discussion

In our study, we assessed energy and macronutrient intake among women undergoing infertility treatment, depending on their body composition. We showed statistically significant differences between the groups (with low, normal, and high fat mass) in terms of protein intake. Its consumption per kg of body weight in this group was the lowest in women with the highest fat tissue content. The groups also differed significantly in the intake of plant protein, which was the lowest in overweight women, both overall and per kg of body weight.

In our study, nearly one-third (29.4%) of the women were classified as overweight or obese, a condition widely recognized as impacting female fertility through mechanisms such as ovulation disorders, hormonal imbalance, and increased time to conception [12]. Excess body weight has been shown to reduce the effectiveness of infertility treatments, including assisted reproductive technologies, by altering metabolic and hormonal functions [13]. These findings underscore the importance of addressing body composition as part of infertility treatment to potentially enhance reproductive outcomes. In the meta-analysis by Moslehi et al. [14], the BMI value correlated negatively with the values of Anti-Müllerian Hormone, FSH in fertile non-PCOS women and inhibin β. For inhibin β, only two studies were included, so a meta-analysis was not possible. In both cases, the correlation of BMI and inhibin β concentration was negative. This indicates lower ovarian reserve in obese women. In overweight and obese women, there is also an unfavorable impact of high BMI on the quality of oocyte maturation. This is probably related to the metabolic and hormonal activity of adipose tissue, which affects the concentration of sex hormones and the concentration of inflammatory mediators [13]. Women with a higher BMI also have a longer time to pregnancy after giving up contraception. In the study of Burger et al. [15], that was 5.3 months for women with normal BMI and 8.2 months for women with obesity. An additional aggravating factor was the irregularity of the menstrual cycle, which occurs more often in overweight and obese women [13]. In turn, according to Sundaram et al. [16], a female WHR ≥ 0.8 (vs. <0.8) and a waist circumference ≥ 88.6 cm (vs. <80 cm) were also responsible for reduced fertility.

It is well-documented that reducing body weight by overweight/obese women has a positive impact on their fertility. The systematic review by Hunter et al. [17] confirms the beneficial effect of nutritional intervention on weight loss in comparison with no/minimal intervention or immediate access to ART. The same study also noted an increase in the number of clinical pregnancies and live births when comparing lifestyle intervention and no/minimal intervention. The meta-analysis by Best et al. [18] compared the effectiveness of lifestyle intervention and standard care and confirmed the beneficial effect of diet and exercise on the number of pregnancies and time to conception, which was 7.2 vs. 5.2 months in the lifestyle intervention group and control group, respectively. However, Caldwell et al. [19] assessed the impact of weight loss on the possibility of getting pregnant as ambiguous. They indicate the need to adjust a lifestyle intervention for a specific person. It is also possible that the inconclusive results regarding the impact of weight loss on the chance of getting pregnant are because body weight and BMI are not ideal indicators in this context. For this reason, the present study focused on body composition (mainly fat mass), which reflects metabolic health much better [20].

The results from the American Lifestyle and Fertility Study [21] revealed that a woman’s higher dietary energy density was associated with a longer time to conception. In turn, in our study, we did not observe any significant differences in energy intake between women with different fat mass content, even though the value of resting energy expenditure, estimated by a body composition analyzer, differs significantly between the groups, and was the highest in the group with the highest fat mass content. This may suggest that the amount of energy consumed above the calculated basal requirement is not the only factor determining the accumulation of fat mass. We also know that BMR per kg of body weight, when measured by indirect calorimetry, which determines the actual energy expenditure, is lower for overweight/obese people than for normal weight people [22]. It is also worth noticing that, according to the literature, a high body fat content and high BMI in women are associated with underestimation of the energy intake, when the data are based on self-reported food diaries [23]. However, the results of the meta-analysis by Rouhani et al. [24] did not indicate a clear relationship between the energy density of the diet and body weight or BMI. This association was more pronounced with more weight gain on a high energy density diet compared to a low energy density diet in cohort studies.

In the investigated group, a higher protein intake per kg of body weight was associated with lower body weight, BMI, fat mass, waist and abdominal circumference, WHR, and abdominal fat index. It is well-documented that a higher total protein intake is also associated with a positive effect on insulin secretion and blood glucose levels [25,26]. On the other hand, according to Chavarro et al. [27], a high dietary protein intake was associated with a higher risk of ovulatory infertility. In the mentioned study, women with the highest protein intake had a 41% higher risk of ovulatory infertility than women with the lowest protein intake. Our analysis showed significant differences in protein intake per kilogram of body weight across groups, with women in the high-fat group exhibiting the lowest protein intake relative to body mass. This difference is likely due to their higher overall body weight rather than a reduced absolute intake of protein. Given that total energy intake did not vary significantly between groups, it suggests that women with higher fat percentages may not be consuming enough protein relative to their body composition. In the Polish Nutritional Standards [11], the Recommended Dietary Allowance (RDA) is set at 0.9 g/kg body mass. Also, according to other Polish authors, a high protein supply exceeding the recommended values is associated with a greater risk of infertility [28]. However, there was no significant correlation between anthropometric parameters and total protein intake. These results coincide with the results of a large umbrella review by Ellinger et al. [29], which questioned the relationship between protein intake and parameters such as body weight, body fat, and waist circumference.

In the previously mentioned study of Chavarro et al. [27], a similar relationship occurred between the amount of animal protein consumption and the risk of ovulatory infertility. For the highest animal protein intake, the risk of ovulatory infertility was 39% greater than for the lowest. This effect may be related to greater insulin secretion in response to animal protein and the influence on testosterone concentration in women [6]. Among surveyed women, in both entire sample and individual groups, the animal-to-plant protein ratio was higher than 1:1. In the study by Gorna et al. [28], in both groups of women with infertility and in the control group, the consumption of animal protein was higher than the consumption of plant protein. This may suggest that such a tendency exists in the entire population. In our study, the highest animal-to-plant protein ratio was noticed in women with the highest fat mass content. However, the results of another study indicated that higher consumption of animal protein (per 150 kcal) was associated with weight gain—especially in women [30].

We reported that a high intake of animal protein correlated positively with a high total fat intake. According to Polish recommendations, the amount of dietary energy from fat should not exceed 35% [11].

There are studies suggesting an adverse effect of a high-fat diet on the functioning of the reproductive system, but the exact mechanism of such action is unclear [31]. The quality of the dietary fat is particularly important. For example, a higher intake of trans fatty acids positively correlates with the occurrence of infertility due to ovulation disorders. In the study by Chavarro et al. [32], a 2% higher trans fatty acid (TFA) intake resulted in a 94% increase in the risk of ovulatory infertility. In our study, the groups did not differ significantly in the intake of SFA, MUFA, or polyunsaturated fatty acids (PUFA), but similar to the Chavarro et al. study [27], the consumption of animal protein correlated positively with saturated fatty acids intake. Research shows that high consumption of these fatty acids, in comparison to polyunsaturated fatty acids, is related to a greater increase of total and visceral fat mass, which, as we know from the literature, can promote fertility impairment [33,34].

Significant differences between the three groups were observed in the consumption of plant protein in grams and plant protein in grams per kg of body weight. Women with the lowest body fat content had the highest consumption of plant protein. This relationship was confirmed by studies showing that high consumption of plant protein among women correlates negatively with BMI values and waist circumference [35]. Regarding female fertility, a low intake of plant protein may affect the sex hormone concentration, e.g., luteal phase progesterone and FSH, and increase the frequency of anovulatory cycles in women of reproductive age even with proper protein and energy intake [36]. According to Cavarro et. al. [27], replacing 5% of energy from animal protein with plant protein reduces the risk of ovulatory infertility by 50%.

High plant protein consumption in the study group resulted in higher consumption of PUFA. This group includes omega-3 fatty acids, which thanks to their antioxidant properties, have a positive effect on the ovulation process, the concentration of sex hormones, oocyte quality, embryo implantation, and the menstrual cycle. They also relieve inflammation that can interfere with the proper functioning of reproductive organs [37]. Additionally, they improve insulin sensitivity and lipid profile, which is particularly beneficial for women with polycystic ovary syndrome [6]. According to Guo et al. [38], a higher blood concentration of omega-3 fatty acids was associated with a 37% lower risk of metabolic syndrome, which may be a factor extending the time to get pregnant [39]. In our study, in most cases (75%, *n* = 38), the intake of combined EPA and DHA was below the recommendations of 250 mg/d [11]

An adverse effect on fertility has been noted in diets with a high glycemic index and load due to the increased risk of insulin resistance, lipid disorders, and increased oxidative stress. Insulin resistance and hyperinsulinemia promote ovulation disorders and hyperandrogenism [6]. In our study group, the dietary daily intake of sugar exceeded 5% of total energy in 31 women (61%), and the highest was at the level of 17.7%. It can certainly be claimed that reducing the sugar intake is an important recommendation in this group. In the study group, 62.7% of women had a sugar consumption above the recommended 5% of the energy value of the diet. After division into subgroups, it was 66.7%, 59.3%, and 66.7% for women with low, normal, and high body fat content, respectively. This shows that not only overweight or obese women should pay attention to the amount of sugar in their diet. Scientific evidence shows that the occurrence of diabetes, insulin resistance, and elevated fasting glucose levels are associated with reduced fertility [40]. A study by Zhao et al. [41] on a large group of couples trying to conceive for the first time showed that a higher female fasting glucose concentration is associated with lower fertility. The highest percentage of pregnancies within 12 cycles was recorded in women with normal glucose tolerance (42.29%), lower in women with impaired glucose tolerance (35.52%), and the lowest in women with diabetes (31.52%). Moreover, there was an inverse correlation between glucose levels and fertility even when glucose levels were within the normal range. A diet with a high glycemic index and load is associated with the occurrence of PCOS, ovulation disorders, and menstrual cycle disorders as well as a greater risk of female infertility [42].

The presented research results show trends in the nutrition of women struggling with infertility and indicate the direction in which nutritional education in this group should follow. Nevertheless, this study has several limitations. First, the cross-sectional design precludes any causal inference between dietary composition and infertility outcomes, as all participants were women actively undergoing infertility treatment. Consequently, while associations between dietary intake, body composition, and markers relevant to reproductive health were identified, causative relationships cannot be established. Additionally, the sample size was relatively small and limited to a specific clinical population, which may affect the generalizability of our findings to broader populations. Finally, dietary intake was assessed using self-reported 3-day dietary records, which, despite careful verification by a dietitian, may be subject to recall bias or underreporting.

It is also important to note that the study cohort, consisting of women undergoing infertility treatment, may not be fully representative of the general population of young women. Women seeking infertility treatment may differ from a control group without fertility issues in terms of physiological factors and lifestyle, which could influence the observed relationships between diet composition, body composition, and reproductive health. Literature suggests for example that women experiencing infertility have a high prevalence of polycystic ovary syndrome (PCOS) [43] which is strongly correlated with insulin resistance [44]. It is associated with specific dietary habits that may alter their nutritional needs and impact body composition [45]. Consequently, some of the identified associations may be specific to this clinical population and may not reflect trends observed in women without fertility concerns.

## 5. Conclusions

This study highlights significant associations between dietary composition, body composition, and nutritional status in women undergoing infertility treatment. Specifically, higher protein intake, particularly from plant sources, was associated with lower fat mass and improved body composition metrics. However, as this study was observational, establishing causality is impossible. These findings underscore the importance of individualized nutritional counseling as part of comprehensive infertility care. However, further longitudinal studies are necessary to asses causative relationships between dietary modifications and reproductive health outcomes.

## Figures and Tables

**Table 1:** Age, anthropometric parameters and basal metabolic rate ^1^ among study participants concerning body fat content.

Characteristics	All, *n* = 51	Low Fat (I), *n* = 12	Normal Fat (II), *n* = 27	High Fat (III), *n* = 12
Age (years)	34.0 (32.0–35.0)	32.0 (30.5–34.3)	34.0 (33.0–35.0)	33.5 (31.8–37.3)
Weight (kg)	61.5 (54.8–72.7)	49.8 (47.1–53.1)	61.5 (58.1–65.9)	87.6 (79.5–96.0)
Height (cm)	166.0 (160.5–170.0)	160.5 (158.8–165.3)	168.0 (164.0–170.0)	167.0 (163.0–173.0)
BMI (kg/m^2^)	22.2 (20.2–25.5)	18.7 (18.3–19.0)	21.9 (21.3–23.6)	31.7 (27.2–34.4)
Fat mass (%)	26.1 (22.6–32.5)	16.9 (15.4–20.0)	26.1 (25.1–29.2)	40.4 (35.9–44.7)
Visceral fat (level)	3.0 (2.0–4.0)	1 (1–1)	3 (2–3)	8 (5.75–9.25)
Total body water (%)	51.5 (47.8–53.7)	57.2 (55.575–58.075)	51.5 (49.25–52.3)	42.85 (40.2–45.625)
Muscle mass (kg)	42.3 (40.2–46.8)	39.5 (37.4–40.775)	42.2 (40.85–46)	49.75 (46.9–52.825)
Muscle mass (%)	70.1 (64.0–73.4)	78.9 (76.1–80.3)	70.1 (67.2–71.1)	56.6 (52.5–60.8)
Waist circumference (cm)	73 (68.0–83.5)	64.5 (63.5–67)	73 (70.5–76.5)	97.5 (88.5–107.8)
Hip circumference (cm)	98 (93.5–103)	90 (89–92.25)	98 (95.5–100)	112 (106.5–120)
WHR	0.76 (0.72–0.80)	0.715 (0.697–0.742)	0.76 (0.73–0.78)	0.895 (0.8275–0.925)
Basal metabolic rate (kcal)	1341 (1270.5–1496.5)	1236.5 (1174.5–1280)	1341 (1291.5–1451)	1601.5 (1539.25–1723)

^1^ All values are presented as median and quartile range. BMI—body mass index. WHR—waist–hip-ratio.

**Table 2:** Demographic and anamnestic characteristics of the group.

Variable		Frequency, *n* (%)
Education level	Secondary school	4 (7.8)
	Bachelor’s degree	6 (11.8)
	Master’s degree	39 (76.5)
	Doctoral degree	2 (3.9)
Duration of infertility	≤3 years	25 (49.0)
	>3 years	13 (25.5)
	NA	13 (25.5)
Chronic diseases/conditions	Endometriosis	2 (3.9)
	Polycystic ovary syndrome	5 (9.8)
	Hypothyroidism	10 (19.6)
	Insulin resistance	8 (15.7)
Supplementation	Multivitamin	14 (27.5)
	Folic acid	29 (56.9)
	Vitamin D	34 (66.7)
	Omega-3 fatty acids	10 (19.6)
	Myo-inositol	8 (15.7)
	Magnesium	9 (17.6)
Special diets	Vegetarian	4 (7.8)
	Low-carbohydrate diet	4 (7.8)
Physical activity	≥150 min weekly	14 (27.5)
	<150 min weekly	25 (49.0)
	No additional activity	12 (23.5)

NA—not available.

**Table 3:** Intake of energy and selected nutrients.

Intake ^1^	All, *n* = 51	Low Fat (I) (*n* = 12)	Normal Fat (II) (*n* = 27)	High Fat (III) (*n* = 12)	*p* ^2^
Energy intake (kcal)	1675 (1472–1990)	1613 (1459–1824)	1705 (1472–1951)	1704 (1482–2028)	0.952 ^
BMR/kcal intake (%)	81.5 (71.3–96.3)	72.3 (61.2–84.9)	78.4 (69.3–97.2)	95.4 (83.4–102.0)	0.003 ^
Protein intake (g)	69.2 (62.5–81.8)	77.1 (66.8–84.0)	65.0 (62.2–79.2)	69.8 (63.3–107.5)	0.548 #
Protein intake (g/kg mc)	1.1 (0.9–1.4)	1.5 (1.4–1.6)	1.1 (1–1.3)	0.8 (0.8–1.0)	<0.001 *#
Animal protein (g)	40.3 (34.5–55.4)	42.8 (21.0–49.4)	39.7 (34–55.4)	42.5 (34.9–76.1)	0.712 #
Plant protein (g)	27.8 (23.3–34.0)	34.3 (27.4–38.3)	26.6 (23.2–29.7)	27.6 (22.6–31.0)	0.030 *^
Plant protein (g/kg)	0.43 (0.34–0.56)	0.67 (0.57–0.76)	0.44 (0.38–0.48)	0.30 (0.27–0.35)	<0.001 *^
Animal: plant protein ratio	1.6 (1.1–2.3)	1.3 (0.6–1.9)	1.6 (1.2–2.3)	1.9 (1.6–2.2)	0.187 #
Fat intake (g)	56.7 (46.7–68.6)	58.3 (48.5–69.1)	56.0 (46.7–64.9)	57.9 (53.3–71.2)	0.939 #
Fat intake (% of energy)	30.7 (26.6–34.8)	30.7 (27.3–35.8)	30.5 (27.7–34.0)	32.2 (26.2–34.6)	0.942 ^
SFA (g)	19.2 (15.7–27.6)	16.4 (13.1–23.4)	19.2 (17.2–27.7)	21.1 (18.4–27.1)	0.442 ^
MUFA (g)	23.8 (17.6–28.4)	24.4 (17.6–28.3)	23.2 (18.3–26.9)	24.3 (19.1–30.5)	0.984 ^
PUFA (g)	9.5 (7.7–10.9)	10.5 (8.5–14.2)	9.5 (7.6–10.4)	9.2 (7.3–10.8)	0.370 #
EPA + DHA (mg)	63.7 (33.3–339.8)	61.4 (33.7–223.7)	57.4 (32.4–147.3)	123.4 (34.8–502.8)	0.764 #
Carbohydrates (g)	216.4 (185–256)	221.5 (185.7–261.5)	216.0 (184.2–259.5)	234.8 (188.7–252.6)	0.943 ^
Carbohydrates (% of energy)	52.8 (46.1–55.6)	52.8 (46.0–56.5)	53.0 (46.6–55.7)	49.3 (46.8–54.2)	0.917 ^
Sugars (% of energy)	5.7 (4.4–8.0)	6.2 (4.6–8.3)	5.7 (4.3–7.7)	5.6 (4.6–8.0)	0.852 #
Dietary fiber (g)	19.9 (16–26.2)	27.5 (20.8–30.7)	18.6 (15.05–25.0)	20.3 (17.1–25.8)	0.010 *^

^1^ Presented as median and quartile range. ^2^ *p*-values obtained by ANOVA (normal distribution ^) and KRUSKAL (non-normal distribution #), * statistically significant (*p* < 0.05), BMR—basal metabolic rate. SFA—saturated fatty acids, MUFA—monounsaturated fatty acids, PUFA—polyunsaturated fatty acids. EPA—eicosapentaenoic acid DHA—docosahexaenoic acid.

**Table 4:** Nutrient intake characteristics according to Polish Nutritional Standards.

Nutrient	Intake Below Recommendation, *n* (%)	Intake Above Recommendation, *n* (%)	Polish Nutritional Standards [10]
Protein	7 (13.7)	44 (86.3)	0.9 g/kg body mass
Fat	1 (2.0)	12 (23.5)	20–35% of energy
Sugar	NA	32 (62.7)	≤5% of energy
EPA + DHA	38 (74.5)	ND	≥250 mg/d
SFA	ND	32 (62.7)	≤10%
Dietary fiber	33 (64.7)	ND	≥25 g/d

SFA—saturated fatty acids, EPA—eicosapentaenoic acid, DHA—docosahexaenoic acid, ND—no data.

## Data Availability

The raw data supporting the results and conclusions of this article will be made available by the authors on request. The data are not publicly available due to privacy restrictions.
